# Synergistic Antibacterial Effects of Meropenem in Combination with Aminoglycosides against Carbapenem-Resistant *Escherichia coli* Harboring *bla*_NDM-1_ and *bla*_NDM-5_

**DOI:** 10.3390/antibiotics10081023

**Published:** 2021-08-23

**Authors:** Pawarisa Terbtothakun, Ozioma Forstinus Nwabor, Thanyaluck Siriyong, Supayang P. Voravuthikunchai, Sarunyou Chusri

**Affiliations:** 1 Division of Biological Science, Faculty of Science and Natural Product Research Center of Excellence, Prince of Songkla University, Hat Yai 90110, Songkhla, Thailand; pawarisa.3tk@gmail.com (P.T.); supayang.v@psu.ac.th (S.P.V.); 2 Division of Infectious Diseases, Department of Internal Medicine, Faculty of Medicine, Prince of Songkla University, Hat Yai 90110, Songkhla, Thailand; nwaborozed@gmail.com; 3 Traditional Thai Medical Research and Innovation Center, Faculty of Traditional Thai Medicine, Prince of Songkla University, Hat Yai 90110, Songkhla, Thailand; thanyaluck.s@psu.ac.th

**Keywords:** aminoglycosides, antibiotic synergism, carbapenem-resistant *Escherichia coli*, combination therapy, ESBL genes

## Abstract

Infections due to carbapenem-resistant *Escherichia coli* (CREC) are problematic due to limitation in treatment options. Combination therapies of existing antimicrobial agents have become a reliable strategy to control these infections. In this study, the synergistic effects of meropenem in combination with aminoglycosides were assessed by checkerboard and time-kill assays. Of the 35 isolates, 19 isolates (54.3%) were resistant to carbapenems (imipenem and meropenem) with the MIC ranges from 16 to 128 µg/mL. These isolates were resistant to almost all antibiotic classes. Molecular characteristics revealed co-harboring of carbapenemase (*bla*_NDM-1_, *bla*_NDM-5_ and *bla*_OXA-48_) and extended-spectrum β-lactamases (ESBL) genes (*bla*_CTX-M_, *bla*_SHV_ and *bla*_TEM_). The checkerboard assay displayed synergistic effects of meropenem and several aminoglycosides against most CREC isolates. Time-kill assays further demonstrated strong synergistic effects of meropenem in combination with either amikacin, gentamicin, kanamycin, streptomycin, and tobramycin. The results suggested that meropenem in combination with aminoglycoside therapy might be an efficient optional treatment for infections cause by CREC.

## 1. Introduction

Infections due to carbapenem-resistant *Escherichia coli* (CREC), particularly the New Delhi metallo-β-lactamases (NDM)-producing isolates, are critically problematic to global health care [1]. These infections usually yield unfavorable clinical outcomes, prolonged length of hospitalization and high hospital costs [2]. The national antimicrobial resistance surveillance data reported by the Thailand National Institute of Health (2016–2018), indicated a high prevalence of carbapenem-resistant *Enterobacteriaceae* (CRE) (93%) among hospitalized patients in Thailand [3]. In the past, carbapenems were the most reliable antimicrobial agents against hospital-acquired infections caused by extended-spectrum β-lactamase (ESBL)-producing *Enterobacteriaceae* [4]. However, extensive usage as both empirical and definitive regimens [5], resulted in the emergence of CRE [4].

*Enterobacteriaceae* resistance to carbapenems is mainly associated with the production of several kinds of carbapenemases, which are enzymes capable of hydrolyzing carbapenems and other β-lactams [6]. In addition, the lack of porin proteins by alteration in the permeability of the bacterial cell membrane, and overexpression of efflux pumps are additive carbapenem resistance mechanisms [7]. Numerous epidemiological studies have suggested that the acquisition of carbapenemase-encoding genes might lead to a rapid outbreak mostly in the hospital-setting and sometimes in the community-setting [8,9,10]. Moreover, the specific class of the carbapenemase should be considered during the development of novel antimicrobial agents as each class possesses a unique mechanism and spectrum of activity [11]. Previous studies have reported that ceftazidime-avibactam binds reversibly to class A, C, and some D β-lactamases [12,13], whereas imipenem-cilastatin-relebactam and meropenem-vaborbactam reversibly and competitively inhibited class A and C β-lactamases [14,15]. However, these antibiotics did not inhibit metallo-β-lactamases such as NDM carbapenemases [12,14,15]. Globally, the predominant carbapenemases include NDM, *Klebsiella pneumoniae* carbapenemase (KPC), Verona integrin-encoded metallo-β-lactamase (VIM), imipenemase (IMP), and oxacillinases (OXA)-type enzymes, which are encoded by *bla*_NDM_, *bla*_KPC_, *bla*_VIM_, *bla*_IMP_, and *bla*_OXA_ genes, respectively [6]. However, *bla*_NDM_ has gained relevance due to the high-level of resistance to many clinically available β-lactams and ease of horizontal transfer between different isolates. To date, several variants of NDM enzymes have been identified [16] with amino acid substitutions at different positions. NDM-5 differed from NDM-1 by substitutions at positions 88 (Val→Leu) and 154 (Met→Leu), and several studies have showed that *bla*_NDM-5_ is carried by conjugatable IncX3 plasmids responsible for the rapid spread [17,18,19].

Currently, therapeutic options for the management of infections caused by CREC are limited [20]. Moreover, the development of new antimicrobial agents are costly, time-consuming, and require various stages of toxicological evaluations to ensure safety [11]. Hence, combining existing antimicrobial agents has become a strategy against several kinds of infections caused by multi-drug resistant (MDR) organisms [21]. Previous studies have supported the use of combination therapy as an effective treatment option for infections caused by several MDR Gram-negative bacteria [22,23,24]. A recent study demonstrated the synergistic effect of meropenem and aminoglycosides against KPC-2 and NDM-1-producing carbapenem-resistant *Klebsiella pneumoniae* [25]. Additionally, the ability of meropenem to potentiate aminoglycoside activity, largely dependent on the MexXY-OprM multidrug efflux system, has been shown [26]. However, data for combinations between meropenem and several aminoglycosides against CREC harboring *bla*_NDM_ genes is lacking. This study evaluated the effects of meropenem in combination with several commonly used aminoglycosides (amikacin, gentamicin, kanamycin, streptomycin, and tobramycin) on CREC isolates harboring *bla*_NDM_ genes.

## 2. Results and Discussion

### 2.1. Bacterial Isolates

A total of 35 suspected CREC isolates were collected from eight hospitals located in Southern Thailand. The isolates were obtained from various clinical specimens, including blood (*n* = 11), rectal (*n* = 19), throat (*n* = 3) and environment (*n* = 2). Data of isolates and antimicrobial response to imipenem and meropenem are shown in Supplementary Materials Table S1. The results indicated that 19 isolates were resistant to carbapenems. Demographic information, clinical data and outcomes of the patients infected with CREC are presented in Table S2. Similar to previous reports of risk factors associated with CRE acquisition or infection [27,28], most of patients in this study had previous exposure to various antimicrobial agents, particularly carbapenems. The results support previous observation that exposure to antibiotics including β-lactams such as carbapenems and cephalosporins, as well as fluoroquinolones were associated with CRE [23]. Patient information indicated that most of the patients were admitted in intensive care units (ICU), which are in consonance with observations of a previous study that showed high prevalence of carbapenemase producing Enterobacteriaceae in the ICU [29].

### 2.2. The Antibiogram of Carbapenem-Resistant E. coli Isolates

The susceptibility profile of CREC isolates was evaluated against 15 conventional antibiotics including carbapenems (imipenem and meropenem), aminoglycosides (amikacin, gentamicin, kanamycin, streptomycin, and tobramycin), cefoperazone-sulbactam, ceftolozane-tazobactam, colistin, cephalosporins (cefotaxime and ceftazidime), fosfomycin, and glycylcyclines (minocycline and tigecycline). The MICs of antibiotics except carbapenems and aminoglycosides were recorded in Table S3 and summarized in Table 1. The results suggested that three antibiotics including colistin, fosfomycin, and amikacin were effective against CREC isolates, with percentage efficacy of 100%, 89.47% and 73.7%, respectively.

To date, polymyxins, fosfomycin, aminoglycosides, and tigecycline are considered choice drugs for the management of infections caused by carbapenem-resistant Gram-negative bacteria [30]. However, resistance to these antibiotics is increasing rapidly with high chance of toxicity due to the relative high doses required for monotherapy medications. Results of this study revealed that approximately 79% of CREC isolates were resistant to tigecycline, contrary to previous reports of 0.7% and 11.2% [31,32]. In addition, the low plasma levels of tigecycline [33] constitutes a clinical concern for mono-therapeutic administration. Polymyxin on the other hand showed excellent antimicrobial effects against CREC with a 100% susceptibility. However, the nephrotoxicity and poor tissue perfusion of polymyxins [34] are limiting factors hindering extensive therapeutic usage. The rapid acquisition of resistance and sodium overload with intravenous fosfomycin [35] are also of clinical concern.

### 2.3. Antimicrobial Susceptibility to Carbapenem and Aminoglycosides

The MIC of carbapenems and aminoglycosides on 19 CREC isolates were determined by the broth microdilution method (Table 2) The 19 isolates were resistant to imipenem (MIC_50_ = 64 µg/mL and MIC_90_ = 128 µg/mL), meropenem (MIC_50_ = 128 µg/mL and MIC_90_ = 128 µg/mL), and streptomycin (MIC_50_ = 512 µg/mL and MIC_90_ = 1024 µg/mL). In addition, 16 isolates were resistant to tobramycin (MIC_50_ = 32 µg/mL and MIC_90_ > 1024 µg/mL), while two isolates were intermediate. Furthermore, 14 and 15 isolates displayed resistance against gentamicin (MIC_50_ = 64 µg/mL and MIC_90_ > 1024 µg/mL) and kanamycin (MIC_50_ = 128 µg/mL and MIC_90_ > 1024 µg/mL), respectively. In contrast, amikacin showed high efficacy on 14 isolates.

Aminoglycosides are an important class of bactericidal antibiotics that are frequently used for the treatment of severe infections caused by Gram-negative bacteria. The major resistance mechanism to aminoglycosides in Gram-negative bacteria is the production of aminoglycoside-modifying enzymes (AMEs) or the modification of ribosome by acquired 16S rRNA methyltransferases (RMTases) [36,37]. AMEs modify select to specific aminoglycosides, hence bacterial isolates show discordant susceptibility among different aminoglycosides.

A previous study demonstrated the co-occurrence of aminoglycoside and β-lactam resistance mechanisms in *E. coli* isolates [38]. In addition, co-harboring of ESBLs, carbapenemases, and 16S rRNA methylase genes within a plasmid have been noted to result in multidrug-resistance in Enterobacteriaceae [39].

### 2.4. Genotypic Resistance Mechanism in Carbapenem-Resistant E. coli Isolates

The 19 CREC isolates were screened for antimicrobial resistance genes including carbapenemase genes (*bla*_KPC_, *bla*_IMP_, *bla*_VIM_, *bla*_NDM_, and *bla*_OXA-48_) and ESBL genes (*bla*_TEM_, *bla*_SHV_, and *bla*_CTX-M_) using PCR (Table 3). The results for carbapenemase genes, demonstrated high prevalence of *bla*_NDM-1_ and *bla*_NDM-5_. However, *bla*_OXA-48_ was observed in one of the tested isolates. Furthermore, co-harboring of carbapenemase and ESBL genes were represented in almost all isolates. The results showed that six isolates with *bla*_NDM-1_ co-harbored *bla*_CTX-M_ and *bla*_TEM_ (Table 2). Additionally, CREC 18 carrying *bla*_NDM-1_ and *bla*_OXA-48,_ co-harbored ESBL genes (*bla*_CTX-M_ and *bla*_TEM_). *bla*_NDM-5_ was found in nine isolates co-harboring ESBL genes (*bla*_CTX-M_ and *bla*_TEM_). However, two out of the nine isolates that harbored *bla*_NDM-5_ had only *bla*_TEM_. The results further showed that three of the isolates had no carbapenemase genes but carried ESBL genes. According to the Ambler classification method, carbapenemase-produced by *Enterobacteriaceae* can be classified into three classes including class A, class B, and class D β-lactamases [6]. However, the clinical relevance of Ambler class C is still unknown [40]. The most widely spread carbapenemase in *E. coli* include class A; KPC, class B; NDM-1, NDM-5, NDM-9, and VIM, class D; OXA-48, OXA-181, and OXA-244 [41,42]. Class A, B and D β-lactamases enzymes are plasmid-mediated and are responsible for the high levels of antimicrobial resistance and rapid dissemination by horizontal transfer [43]. Epidemiological studies have revealed the diversity of carbapenemases predominate in several regions and countries [43]. In the United States, Argentina, Columbia, Greece, Israel, and Italy, KPC-producing *Enterobacteriaceae*, are mostly endemic among nosocomial isolates [1]. NDM was reported as the main carbapenemase-mediating resistance in *E. coli* isolates in India, Pakistan, and Sri Lanka, whereas OXA-48 was reported in North Africa, Malta, and Turkey [44]. NDM and OXA-48 were identified in both nosocomial and community-acquired pathogens [43,45]. A recent study done in Thailand reported a high prevalence (99%) of CREC isolates having at least one carbapenemase-producing gene (CP-gene) [3]. The most common CP-gene among CREC isolates in Thailand were *bla*_NDM_ (94%) and a *bla*_OXA-48-like_ (18%) gene [3]. In this study, *bla*_NDM_ was found in 16 isolates, including seven isolates harboring *bla*_NDM-1_ and nine isolates harboring *bla*_NDM-5_. Similar results were reported in a recent study with a high prevalence of NDM-1 in *E. coli* [46]. The increased usage of antibiotics maybe driving the evolution of NDM-1 variants. M154L amino acid substitution in NDM-5 was the most common substitution in all NDMs variants leading to increase carbapenemase activity [47]. However, a previous study reported that the difference in the activity of NDM-5 and NDM-1 is due to variations in the affinity for zinc [48]. Moreover, V88L amino acid substitution in NDM-5 contribute to lower catalytic activity on imipenem and meropenem [49]. Several studies showed that *bla*_NDM-5_ was carried by IncX3 plasmids which have been shown to be conjugatable and could explain the rapid spread of *bla*_NDM-5_-carrying isolates [50]. However, *bla*_KPC_ which is the most commonly found in the United States [1], was not presented in this study. So far, the prevalence of *bla*_KPC_ in Thailand has remained very low. A previous report indicated a 0.02% (*n* = 12,741) prevalence of *bla*_KPC-13_ among *Enterobacteriaceae* and 1.7% (*n* = 181) among CRE isolates [51], whereas a separate report showed that the prevalence rate of *bla*_KPC-2_ in CRE isolates was 0.13% (*n* = 2245) [52]. Furthermore, the study illustrated the co-existence of carbapenemase and ESBL genes in CREC isolates. Carbapenems were used as first-line antibiotic for treatment of infection caused by extended-spectrum β-lactamase (ESBL)-producing *Enterobacteriaceae*. Thus, the co-harboring of multiple antibiotic resistance genes will promote multi-resistance, which might amount to significant therapeutic concerns.

### 2.5. The Combined Effect of Meropenem and Aminoglycosides

The results of antimicrobial combinations against the 19 CREC isolates are shown in Table 4 and summarized in Table S4. Synergistic effects were observed for meropenem plus gentamicin and meropenem plus streptomycin in 16 (84.2%) isolates, followed by meropenem plus kanamycin and meropenem plus tobramycin in 15 (79%) isolates. Furthermore, synergistic activity was observed in 13 (68.4%) isolates for meropenem plus amikacin. The isolate CREC 11 (*bla*_CTX-M_ and *bla*_TEM_), with high resistance to aminoglycosides, was resistant to all combinations, while isolate CREC 12 (*bla*_NDM-5_, *bla*_CTX-M_ and *bla*_TEM_) was susceptible to meropenem plus amikacin, or gentamicin, or streptomycin combinations. Combination of meropenem plus gentamicin and meropenem plus tobramycin exhibited synergism against CREC 14 (*bla*_NDM-5_ and *bla*_TEM_). The cross resistance of CREC 11 to all the combinations might be due to the cumulative effects of other resistance mechanisms such as overexpression of efflux pump and/or porin with the β-lactamases leading to high level of resistance. However, the results did not reveal an antagonistic effect for the tested combinations.

The results revealed that addition of aminoglycosides as adjunctive therapy to meropenem could restore meropenem activity against CREC isolate harboring *bla*_NDM_. Combination of meropenem and aminoglycosides might promote membrane disruption since aminoglycosides exert disruptive effects on the outer membrane structure by binding with the negatively charged lipopolysaccharides in the outer membrane of Gram-negative bacteria. Thus, the aminoglycoside promotes the permeabilizing effect and enhances the periplasmic target site penetration of other antibiotics such as carbapenems used in combination [55,56]. Meropenem is a safe, well-tolerated, and commonly used as monotherapy or as combination regimens for hospital-acquired infection due to several MDR Gram-negative bacteria [57,58,59]. Similarly, aminoglycosides are effective against Gram-negative aerobic bacteria including resistant *Enterobacteriaceae* [60]. However, aminoglycosides monotherapies can lead to unfavorable clinical outcomes due to rapid emergence of resistance, and nephrotoxicity among patients with prolonged usage of aminoglycosides [61,62].

### 2.6. Time-Kill Assay

**Figure 1:**
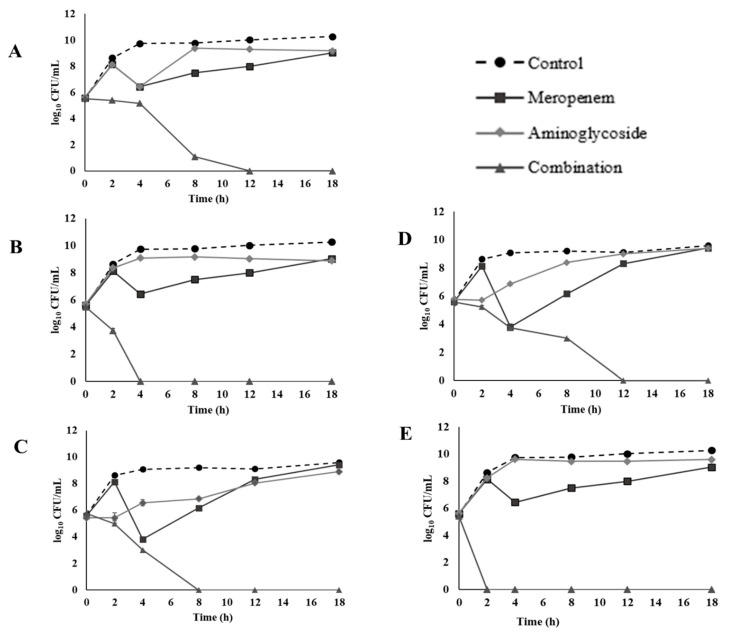
Time-kill curves of 1/4 MIC (32 µg/mL) meropenem and 1/4 MIC aminoglycosides combination against CREC 12: (**A**) amikacin (256 µg/mL), (**B**) gentamicin (256 µg/mL), (**C**) kanamycin (256 µg/mL), (**D**) streptomycin (8 µg/mL), and (**E**) tobramycin (256 µg/mL).

The time-kill effects of meropenem combined with either amikacin, gentamicin, kanamycin, streptomycin, or tobramycin were evaluated on CREC 12 (Figure 1). The results revealed a synergistic bactericidal effect at 1/4 meropenem plus 1/4 amikacin at 4 h. (Figure 1A) and 1/4 meropenem plus 1/4 gentamicin at 2 h. (Figure 1B) with a ≥3 log_10_ CFU/mL reduction in cell growth when compared to the MIC of individual antibiotics. Furthermore, an indifferent effect was revealed at 1/4 meropenem plus 1/4 kanamycin (Figure 1C). At 12 h, combination between 1/4 meropenem plus 1/4 streptomycin (Figure 1D) presented a synergistic bactericidal effect, while combination of 1/4 meropenem plus 1/4 Tobramycin revealed a synergistic effect (Figure 1E).

**Figure 2:**
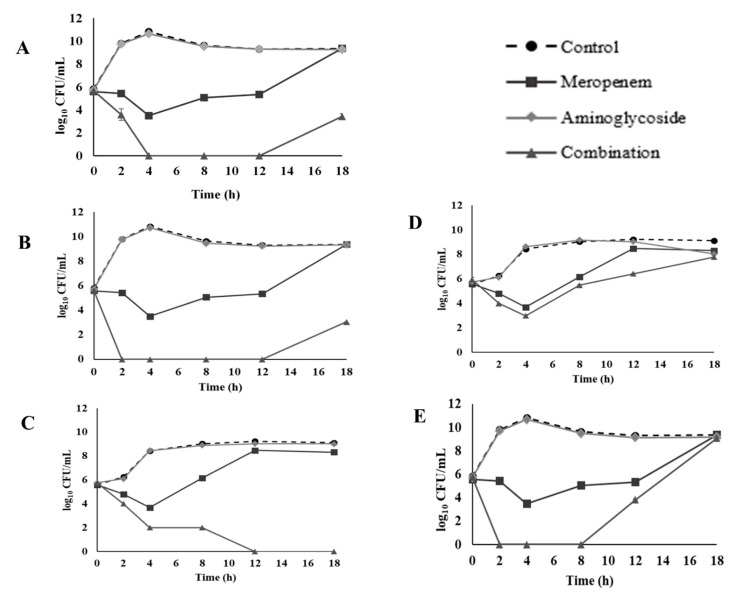
Time-kill curves of 1/4 MIC (32 µg/mL) meropenem and 1/4 MIC aminoglycosides combination against CREC 18: (**A**) amikacin (1 µg/mL), (**B**) gentamicin (16 µg/mL), (**C**) kanamycin (32 µg/mL), (**D**) streptomycin (64 µg/mL), and (**E**) tobramycin (8 µg/mL).

For CREC 18 at 8 h, 1/4 meropenem plus 1/4 amikacin showed a synergistic bactericidal effect (Figure 2A). Similar results were observed at 4 h with 1/4 meropenem plus 1/4 gentamicin (Figure 2B), at 8 h for 1/4 meropenem plus 1/4 kanamycin (Figure 2C), or 1/4 streptomycin (Figure 2D), and at 2 h for 1/4 meropenem plus 1/4 tobramycin against isolate CREC 18 (Figure 2E). However, a regrowth was observed at 8 h for meropenem and tobramycin combination, and at 12 h for meropenem and amikacin or gentamicin combination. Our results showed inconsistencies between the FICI, and time kill methods. Similar findings have been reported by previous studies [63,64].

## 3. Materials and Methods

### 3.1. Chemical and Media

All culture media were purchased from Becton Dickinson & Co. Difco TM (Franklin Lakes, NJ, USA). Colistin sulfate, minocycline hydrochloride, and tobramycin were obtained from Sigma-Aldrich, (Saint Louis, MO, USA). Amikacin, ciprofloxacin, cefotaxime, gentamicin, kanamycin, levofloxacin, and streptomycin were purchased from Siam Bheasach Co, Ltd. (Bangkok, Thailand). Tigecycline was purchased from Pfizer Inc. (Philadelphia, PA, USA). Ceftazidime was obtained from Reyoung Pharmaceutical Co., Ltd. (Shandong, China). Imipenem was obtained from Merck Sharp & Dohme Corp. (Elkton, VA, USA). Meropenem was obtained from M&H Manufacturing Co. Ltd. (Samutprakarn, Thailand). Cefoperazone/sulbactam was obtained from L.B.S. Laboratory Ltd. (Bangkok, Thailand). Ceftolozane/tazobactam was obtained from Steri-Pharma, LLC (Syracuse, NY, USA). Fosfomycin was obtained from Meiji Seika Kaisha, Ltd. (Tokyo, Japan).

### 3.2. Bacterial Collection and Identification

A total of 35 suspected CREC isolates were collected from eight hospitals located in Southern Thailand. The isolates grew on MacConkey agar supplemented with imipenem at 6 µg/mL. All isolates were identified to species level using standard biochemical tests and MALDI-TOF-MS. *E. coli* ATCC 25922 was used as quality control. The samples were kept in tryptic soy broth supplemented with 20% glycerol and stored at −80 °C.

### 3.3. Screening for Carbapenem Resistance

Resistance of the 35 suspected CREC isolates was assessed by the broth microdilution method according to the Clinical and Laboratory Standards Institute [65]. Briefly, the isolates were grown in cation-adjusted Mueller–Hinton broth (CAMHB). Bacterial cultures were adjusted with sterile 0.85% NaCl to McFarland 0.5 turbidity standard. Aliquot of 100 μL diluted bacterial suspension (1 × 10^6^ CFU/mL) was mixed with 100 μL antibiotic in a 96-well plate and incubated at 37 °C for 18 h. The minimum inhibitory concentration (MIC) was expressed as the lowest concentration of the antibiotic that inhibits visible growth after incubation as indicated by the resazurin test.

### 3.4. Antibiogram of Carbapenem-Resistant Isolates

Confirmed CREC isolates were exposed to 17 conventional antibiotics including carbapenem (imipenem and meropenem), aminoglycosides (amikacin, gentamicin, kanamycin, streptomycin, and tobramycin), cefoperazone-sulbactam, ceftolozane-tazobactam, cephalosporins (cefotaxime and ceftazidime), colistin, fluoroquinolone (ciprofloxacin and levofloxacin), fosfomycin, glycylcyclines (minocycline and tigecycline). The MICs of the antibiotics were determined using the broth microdilution method as previously detailed. The MIC for fosfomycin, was determined by the agar dilution method. Briefly, cation-adjusted Mueller–Hinton agar (CAMHA) was supplemented with 25 mg/L glucose-6-phosphate (G6P) as recommended by CLSI guidelines [65]. The bacterial suspension (approximately 1 × 10^4^ CFU/mL) was spotted at 10 microliters on the surface of each agar plate containing the antibiotic.

### 3.5. Genotypic Determination of Carbapenemase and ESBL

Genomic DNA from *E. coli* was prepared using Presto^TM^ Mini gDNA Bacteria Kit. Quantification of the extracted DNA was determined by spectroscopy at 260 nm. Antimicrobial resistance genes, including carbapenemase (*bla*_IMP_, *bla*_KPC_, *bla*_NDM_, *bla*_OXA-48_, and *bla*_VIM_) and ESBL (*bla*_CTX-M_, *bla*_SHV_, and *bla*_TEM_) were detected by PCR using the primers shown in Table 3. The amplification conditions for detecting IMP, KPC, and OXA-48 genes were initial denaturation at 94 °C for 10 m, 36 cycles of 94 °C for 30 s, 52 °C for 40 s, and 72 °C for 50 s, and final elongation at 72 °C for 5 m. The amplification condition for NDM and VIM genes were initial denaturation at 94 °C for 10 m, 36 cycles of 94 °C for 30 s, 56 °C for 40 s, and 72 °C for 50 s, and final elongation at 72 °C for 5 m. The amplification conditions for detecting ESBL genes included CTX-M, SHV, and TEM genes were initial denaturation at 95 °C for 15 m, 30 cycles of 94 °C for 30 s, 60 °C for 30 s, and 72 °C for 2 m, and final elongation at 72 °C for 10 m.

### 3.6. Checkerboard Technique

The synergistic activities of meropenem combined with five aminoglycosides (amikacin, gentamicin, kanamycin, streptomycin, and tobramycin) on CREC were determined by the checkerboard technique. Briefly, 100 µL of 1 × 10^6^ CFU/mL bacterial suspension was added to wells containing 50 µL of each subinhibitory concentrations of meropenem and aminoglycosides. The plates were incubated for 18 h at 37 °C. Inhibitory concentrations were determined as concentrations without bacterial growth as indicated by the resazurin test. The experiments were performed in triplicate for three independent repeats. The activity of the antimicrobial combinations was defined by the fractional inhibitory concentration index (FICI), as follows:

(1) $$\mathrm{FICI}\;=\frac{\mathrm{MIC}\;\mathrm{of}\;\mathrm{drug}\;A\;\mathrm{in}\;\mathrm{combination}}{\mathrm{MIC}\;\mathrm{of}\;\mathrm{drug}\;A\;\mathrm{alone}}\;+\;\frac{\mathrm{MIC}\;\mathrm{of}\;\mathrm{drug}\;B\;\mathrm{in}\;\mathrm{combination}}{\mathrm{MIC}\;\mathrm{of}\;\mathrm{drug}\;B\;\mathrm{alone}}$$

FICI results for each combination were interpreted as follows: FICI ≤ 0.5, synergism; 0.5 < FICI ≤ 4, indifference; and FICI > 4, antagonism. *E. coli* ATCC 25922 was used as standard control strains for the assays [66].

### 3.7. Time-Kill Assay

The activity of meropenem and aminoglycosides combinations were confirmed by the time-kill assay. Antibiotics were tested alone and in combination at 1/4 MIC. An inoculum size of 1 × 10^6^ CFU/mL was added and incubated at 37 °C. Bacterial growth controls were maintained throughout the experiment. Bacterial growth was assessed at 0, 2, 4, 8, 12 and 18 h by plating 10-fold serially diluted suspensions on Mueller–Hinton agar plates. Plates were incubated overnight at 37 °C, and the number of colonies were counted. The experiments were performed in triplicate and recorded as mean averages. Bactericidal activity was defined as a ≥3 log_10_ CFU/mL reduction when compare the number of viable cells at time zero (0 h). Antibiotic combination synergism was defined as a ≥2 log_10_ CFU/mL at 18 h for the antimicrobial combination, compared with the most active agent. Indifferent was defined as <2 log_10_ CFU/mL increase or decrease at 18 h for the drug combination when compare with the most active drug and antagonism was defined as ≥2 log_10_ CFU/mL increase between the combination and the most active single drug [67].

## 4. Conclusions

Combination therapies have been highlighted as a possible treatment option for the management of infections caused by drug resistant bacterial isolates. This study demonstrated that combinations of meropenem with aminoglycoside might still be an efficient therapeutic option for the treatment of CREC harboring *bla*_NDM-1_ and *bla*_NDM-5_. However, due to indifferent results observed with the FICI, it is important to consider other mechanisms of aminoglycoside and carbapenem co-resistance. In addition, further studies on toxicology, pharmacokinetics and pharmacodynamics of these combination regimens are required prior to clinical trials.

## Figures and Tables

**Table 1:** Summary of antimicrobial susceptibility of 19 carbapenem-resistant isolates.

Antibiotics	MIC (µg/mL)			Percentage %		
	Range	MIC_50_	MIC_90_	Susceptible	Intermediate	Resistant
Aminoglycoside						
Amikacin	2–> 1024	4	>1024	73.7	0	26.3
Gentamicin	1–> 1024	64	>1024	21	5.3	73.7
Kanamycin	8–> 1024	128	>1024	21	0	79
Streptomycin	16–1024	512	1024	0	0	100
Tobramycin	1–> 1024	32	>1024	5.3	10.5	84.2
β-lactam + β-lactamase inhibitor						
Cefoperazone-sulbactam	256–> 1024	512	>1024	5.3	0	94.7
Ceftolozane-tazobactam	1024–> 1024	>1024	>1024	5.3	0	94.7
Carbapenem						
Imipenem	16–128	64	128	0	0	100
Meropenem	32–128	128	128	0	0	100
Cephalosporin						
Cefotaxime	256–> 1024	>1024	>1024	0	0	100
Ceftazidime	1024–> 1024	>1024	>1024	0	0	100
Fluoroquinolone						
Ciprofloxacin	0.5–512	128	256	5.3	10.5	84.2
Levofloxacin	<0.5–64	16	32	26.3	0	73.7
Glycylcycline						
Minocycline	<2–16	<2	16	68.4	15.8	15.8
Tigecycline	0.0625–4	2	4	21	0	79
Other						
Colistin	0.25–2	0.5	2	100	0	0
Fosfomycin	16–1024	16	1024	89.5	0	10.5

**Table 2:** Antibacterial profile of aminoglycoside and carbapenem resistance in 19 carbapenem-resistant *Escherichia coli* isolates.

Clinical Isolate	Source	*bla* Genotype		MIC (µg/mL)						
				Carbapenem		Aminoglycoside				
		Carbapenemase	ESBL	Imipenem	Meropenem	Amikacin	Gentamicin	Kanamycin	Streptomycin	Tobramycin
CREC 1	Rectal	*bla*_NDM-1_	*bla*_CTX__-M_, *bla*_TEM_	64 (R)	64 (R)	2 (S)	64 (R)	16 (S)	1024 (R)	16 (R)
CREC 2	Rectal	*bla*_NDM-1_	*bla*_CTX__-M_, *bla*_TEM_	64 (R)	64 (R)	2 (S)	64 (R)	16 (S)	1024 (R)	8 (I)
CREC 3	Rectal	-	*bla*_CTX__-M_, *bla*_SHV_, *bla*_TEM_	128 (R)	128 (R)	64 (R)	64 (R)	64 (R)	256 (R)	128 (R)
CREC 4	Throat	*bla*_NDM-5_	*bla*_CTX__-M_, *bla*_TEM_	64 (R)	64 (R)	4 (S)	32 (R)	64 (R)	512 (R)	32 (R)
CREC 5	Rectal	*bla*_NDM-5_	*bla*_CTX__-M_, *bla*_TEM_	64 (R)	64 (R)	4 (S)	64 (R)	64 (R)	512 (R)	32 (R)
CREC 6	Rectal	*bla*_NDM-5_	*bla*_CTX__-M_, *bla*_TEM_	32 (R)	64 (R)	4 (S)	1 (S)	32 (S)	64 (R)	8 (I)
CREC 7	Throat	*bla*_NDM-1_	*bla*_CTX__-M_, *bla*_TEM_	128 (R)	128 (R)	8 (S)	128 (R)	256 (R)	512 (R)	64 (R)
CREC 8	Rectal	*bla*_NDM-1_	*bla*_CTX__-M_, *bla*_TEM_	128 (R)	128 (R)	8 (S)	128 (R)	128 (R)	16 (R)	64 (R)
CREC 9	Environment	*bla*_NDM-1_	*bla*_CTX__-M_, *bla*_TEM_	64 (R)	128 (R)	8 (S)	64 (R)	512 (R)	512 (R)	64 (R)
CREC 10	Rectal	-	*bla*_TEM_	64 (R)	128 (R)	4 (S)	0.5 (S)	8 (S)	32 (R)	1 (S)
CREC 11	Blood	-	*bla*_CTX__-M_, *bla*_TEM_	32 (R)	64 (R)	>1024 (R)	>1024 (R)	>1024 (R)	32 (R)	>1024 (R)
CREC 12	Blood	*bla*_NDM-5_	*bla*_CTX__-M_, *bla*_TEM_	64 (R)	128 (R)	>1024 (R)	>1024 (R)	>1024 (R)	32 (R)	>1024 (R)
CREC 13	Blood	*bla*_NDM-5_	*bla*_CTX__-M_, *bla*_TEM_	32 (R)	128 (R)	8 (S)	1 (S)	128 (R)	32 (R)	32 (R)
CREC 14	Blood	*bla*_NDM-5_	*bla*_TEM_	16 (R)	32 (R)	>1024 (R)	>1024 (R)	>1024 (R)	1024 (R)	512 (R)
CREC 15	Blood	*bla*_NDM-5_	*bla*_CTX__-M_, *bla*_TEM_	64 (R)	64 (R)	4 (S)	1 (S)	128 (R)	512 (R)	16 (R)
CREC 16	Blood	*bla*_NDM-5_	*bla*_CTX__-M_, *bla*_TEM_	64 (R)	128 (R)	2 (S)	128 (R)	128 (R)	512 (R)	16 (R)
CREC 17	Blood	*bla*_NDM-5_	*bla*_TEM_	64 (R)	128 (R)	4 (S)	64 (R)	64 (R)	256 (R)	16 (R)
CREC 18	Blood	*bla*_NDM__-1_, *bla*_OXA__-48_	*bla*_CTX__-M_, *bla*_TEM_	64 (R)	128 (R)	4 (S)	64 (R)	128 (R)	256 (R)	32 (R)
CREC 19	Blood	*bla*_NDM-1_	*bla*_CTX__-M_, *bla*_TEM_	128 (R)	32 (R)	128 (R)	8 (I)	1024 (R)	512 (R)	128 (R)

R, resistant; S, susceptible; I, intermediate.

**Table 3:** Primers used for PCR amplification of carbapenemase and ESBL genes.

Primer Name		Sequence (5′ to 3′)	Amplicon Size (bp)	Reference
Carbapenemase				
*bla*_IMP_	IMP-F	GGAATAGAGTGGCTTAAYTCTC	232	[53]
	IMP-R	GGTTTAAYAAAACAACCACC		
*bla*_KPC_	KPC-F	CGTCTAGTTCTGCTGTCTTG	798	
	KPC-R	CTTGTCATCCTTGTTAGGCG		
*bla*_NDM_	NDM-F	GGTTTGGCGATCTGGTTTTC	621	
	NDM-R	CGGAATGGCTCATCACGATC		
*bla*_OXA-48_	OXA-F	GCGTGGTTAAGGATGAACAC	438	
	OXA-R	CATCAAGTTCAACCCAACCG		
*bla*_VIM_	VIM-F	GATGGTGTTTGGTCGCATA	390	
	VIM-R	CGAATGCGCAGCACCAG		
Extended-spectrum β-lactamase				
*bla*_CTX-M_	CTX-M-U1	ATGTGCAGYACCAGTAARGTKATGGC	573	[54]
	CTX-M-U2	TGGGTRAARTARGTSACCAGAAYCAGCGG		
*bla*_SHV_	bla-SHV.SE	ATGCGTTATATTCGCCTGTG	747	
	bla-SHV.AS	TGCTTTGTTATTCGGGCCAA		
*bla*_TEM_	TEM-164.S	TCGCCGCATACACTATTCTCAGAATGA	445	
	TEM-165.AS	ACGCTCACCGGCTCCAGATTTAT		

**Table 4:** Effects of meropenem and aminoglycosides combinations on 19 carbapenem-resistant *Escherichia coli*.

Clinical Isolate	Meropenem + Amikacin		Meropenem + Gentamicin		Meropenem + Kanamycin		Meropenem + Streptomycin		Meropenem + Tobramycin	
	MIC ^a^	ΣFICI	MIC ^a^	ΣFICI	MIC ^a^	ΣFICI	MIC ^a^	ΣFICI	MIC ^a^	ΣFICI
CREC 1	8/0.5	0.38 (S)	2/8	0.16 (S)	8/4	0.38 (S)	1/256	0.27 (S)	4/2	0.19 (S)
CREC 2	16/0.125	0.31 (S)	2/8	0.16 (S)	16/4	0.50 (S)	1/256	0.27 (S)	4/2	0.31 (S)
CREC 3	8/16	0.31 (S)	2/16	0.27 (S)	32/8	0.38 (S)	32/32	0.38 (S)	4/8	0.09 (S)
CREC 4	4/1	0.31 (S)	8/8	0.38 (S)	8/16	0.38 (S)	8/64	0.25 (S)	8/4	0.25 (S)
CREC 5	16/0.25	0.31 (S)	2/8	0.16 (S)	4/16	0.31 (S)	8/64	0.25 (S)	8/4	0.25 (S)
CREC 6	8/2	0.63 (I)	8/0.125	0.25 (S)	4/8	0.31 (S)	8/8	0.25 (S)	2/2	0.28 (S)
CREC 7	8/2	0.31 (S)	8/8	0.13 (S)	8/32	0.19 (S)	4/128	0.28 (S)	8/8	0.19 (S)
CREC 8	2/2	0.27 (S)	2/16	0.14 (S)	32/32	0.50 (S)	128/1	1.02 (S)	8/8	0.19 (S)
CREC 9	4/2	0.28 (S)	16/8	0.25 (S)	8/32	0.19 (S)	8/128	0.31 (S)	16/8	0.25 (S)
CREC 10	4/2	0.53 (I)	4/0.125	0.28 (S)	16/2	0.38 (S)	16/8	0.38 (S)	32/0.5	0.75 (I)
CREC 11	32/32	0.53 (I)	16/512	0.75 (I)	64/8	1.01 (I)	64/8	1.25 (I)	64/1024	2.00 (I)
CREC 12	32/256	0.50 (S)	8/128	0.19 (S)	64/8	0.51 (I)	32/8	0.50 (S)	64/8	0.51 (I)
CREC 13	2/2	0.27 (S)	1/0.5	0.51 (I)	2/32	0.27 (S)	4/16	0.53 (S)	2/8	0.27 (S)
CREC 14	16/32	0.53 (I)	0.5/128	0.14 (S)	16/512	1.00 (I)	1/128	0.16 (I)	4/64	0.25 (S)
CREC 15	16/1	0.38 (S)	8/0.25	0.31 (S)	2/32	0.27 (S)	32/1	0.25 (S)	8/4	0.31 (S)
CREC 16	64/0.25	0.63 (I)	4/16	0.16 (S)	4/32	0.28 (S)	8/128	0.31 (I)	16/2	0.25 (S)
CREC 17	32/2	0.75 (I)	4/8	0.16 (S)	16/16	0.38 (S)	8/64	0.31 (S)	8/8	0.56 (I)
CREC 18	32/1	0.50 (S)	2/16	0.27 (S)	16/32	0.38 (S)	8/64	0.31 (S)	4/8	0.28 (S)
CREC 19	2/32	0.31 (S)	16/2	0.75 (I)	4/512	0.63 (I)	4/128	0.38 (S)	8/32	0.50 (S)

S, synergy; I, indifferent. ^a^ minimum inhibitory concentration of combination of meropenem/aminoglycoside. The FICI results for each combination were interpreted as follows: FICI ≤ 0.5, synergism; 0.5 < FICI ≤ 4, indifference; and FICI > 4, antagonism.

## Data Availability

Data is contained within the article or Supplementary Material.

## Supplementary Materials

The following are available online at https://www.mdpi.com/article/10.3390/antibiotics10081023/s1, Table S1: Screening for carbapenem resistance in 35 suspected carbapenem-resistant *Escherichia coli* isolates, Table S2: Clinical information and outcome of patients in 19 carbapenem-resistant *Escherichia coli* (CREC) isolates, Table S3: Minimum inhibitory concentrations of antimicrobial agents against the 19 carbapenem-resistant *Escherichia coli* isolates, Table S4: Summary of the synergistic effects of meropenem in combination with aminoglycosides against 19 carbapenem-resistant *Escherichia coli.*
