# Supplementary material for: Synergistic Antibacterial Effects of Meropenem in Combination with Aminoglycosides against Carbapenem-Resistant Escherichia coli Harboring blaNDM-1 and blaNDM-5
Source: Antibiotics (Basel). 2021 Aug 23;10(8):1023. doi: 10.3390/antibiotics10081023 (PMC8388987; doi:10.3390/antibiotics10081023)
Supplement: Supplementary file 1 [file antibiotics-10-01023-s001.zip › antibiotics-1299971-supplementary.pdf]

Supplementary Materials

Table S1. Screening for carbapenem resistance in 35 suspected carbapenem-resistant *Escherichia coli* isolates

Clinical isolate	Code	Source of isolation	MIC (µg/ml)	
			Imipenem	Meropenem
EC1	1PSUsep1R/2	Rectal	0.25 (S)	0.25 (S)
EC2	1PSU6R/2	Rectal	0.25 (S)	0.0156 (S)
EC3	2PSU6R/1	Rectal	64 (R)	64 (R)
EC4	2PSU6R/2	Rectal	64 (R)	64 (R)
EC5	1HY4R/2	Rectal	0.25 (S)	0.0156 (S)
EC6	1HY8R	Rectal	128 (R)	128 (R)
EC7	1HY13Th/1	Throat	64 (R)	64 (R)
EC8	1HY13R/1	Rectal	64 (R)	64 (R)
EC9	1SK1R/1	Rectal	0.25 (S)	0.0156 (S)
EC10	2ST1R/1	Rectal	0.25 (S)	0.0156 (S)
EC11	2ST4R/2	Rectal	0.25 (S)	0.0156 (S)
EC12	2ST7R/1	Rectal	0.25 (S)	0.003 (S)
EC13	2ST7R/2	Rectal	0.25 (S)	0.25 (S)
EC14	1PT5R/1	Rectal	32 (R)	64 (R)
EC15	1PA5Th/1	Throat	0.25 (S)	0.0156 (S)
EC16	1PA5E	Environment	0.25 (S)	0.0156 (S)
EC17	1PA21Th/1	Throat	128 (R)	128 (R)
EC18	1PA21R	Rectal	128 (R)	128 (R)
EC19	1PA21E	Environment	64 (R)	128 (R)
EC20	2PA3R/1	Rectal	0.25 (S)	0.0156 (S)
EC21	2PA3R/2	Rectal	0.25 (S)	0.0156 (S)
EC22	2PA7R/1	Rectal	0.5 (S)	0.0156 (S)
EC23	2PA9R/1	Rectal	0.25 (S)	0.0156 (S)
EC24	2PA21R/1	Rectal	64 (R)	128 (R)

EC25	SK018	Blood	32 (R)	64 (R)
EC26	SK019	Blood	0.5 (S)	0.0156 (S)
EC27	SK020	Blood	0.25 (S)	0.0156 (S)
EC28	SK021	Blood	64 (R)	128 (R)
EC29	TR003	Blood	32 (R)	128 (R)
EC30	PT024	Blood	16 (R)	32 (R)
EC31	PT033	Blood	64 (R)	128 (R)
EC32	PT048	Blood	64 (R)	128 (R)
EC33	PT051	Blood	64 (R)	128 (R)
EC34	NT002	Blood	64 (R)	128 (R)
EC35	NT004	Blood	128 (R)	32 (R)

R, resistant; S, susceptible

Table S2. Clinical information and outcome of patients in 19 carbapenem-resistant *Escherichia coli* (CREC) isolates.

Isolate	Code	Hospital	Source of isolation	Sex	Age	Initial ward	Underlying disease	Previous use of antibiotics
CREC1	2PSU6R/1	Songklanagarind	Rectal	M	73	ICU medicine	DM, HTN, DLD, CVA, CAD, CKD	CRO, IMP
CREC2	2PSU6R/2							
CREC3	1HY8R	Hatyai	Rectal	M	63	ICU medicine	HTN, CKD	CRO, MEM
CREC4	1HY13Th/1	Hatyai	Throat	F	59	ICU surgery	DM, CVA, CAD	CRO, ETP
CREC5	1HY13R/1	Hatyai	Rectal					
CREC6	1PT5R/1	Phatthalung	Rectal	M	48	ICU medicine	HTN, DLD, CKD	CRO, CAZ, PIP/TAZ. IMP
CREC7	1PA21Th/1	Pattani	Throat	M	84	General medicine	DLD, CVA, CAD	CAZ, MEM
CREC8	1PA21R	Pattani	Rectal					
CREC9	1PA21E	Pattani	Environment					
CREC10	2PA21R/1	Pattani	Rectal	M	47	General medicine	CAD	CRO, PIP/TAZ
CREC11	SK018	Songkhla	Blood	M	61	General medicine	COPD	CRO, AZM, PIP/TAZ
CREC12	SK021	Songkhla	Blood					
CREC13	TR003	Trang	Blood	F	46	General surgery	HTN, CVA, CKD	CRO, LVX, ETP
CREC14	PT024	Pattani	Blood	F	52	ICU surgery	CKD, COPD	CRO, IMP
CREC15	PT033	Pattani	Blood	M	36	General medicine	HTN, CKD	CAZ, LVX, MEM
CREC16	PT048	Pattani	Blood	M	37	ICU medicine	DM, CKD	CRO, PIP/TAZ, IMP
CREC17	PT051	Pattani	Blood	M	41	ICU surgery	CAD, CKD	LVX, IMP
CREC18	NT002	Naradhiwas Rajanagarindra	Blood	F	65	General surgery	DM	CRO, LVX, IMP

CREC19	NT004	Naradhiwas Rajanagarindra	Blood	F	49	General medicine	HTN, CKD	CRO, MEM

AZM, azithromycin; CAD, coronary artery disease; CAZ, ceftazidime; CKD, chronic kidney disease; COPD, chronic obstructive pulmonary disease; CRO, ceftriaxone; CVA, cerebrovascular disease; DM, diabetes mellitus; DLD, dyslipidemia; ETP, ertapenem; HTN, hypertension; IMP, imipenem; LVX, levofloxacin; MEM, meropenem; PIP/TAZ, piperacillin/tazobactam.

Table S3. Minimum inhibitory concentrations of antimicrobial agents against the 19 carbapenem-resistant *Escherichia coli* isolates

Clinical isolate	**Minimum inhibitory concentration (μg/ml)**									
	β-lactam + β-lactamase inhibitor		Cephalosporins		Fluoroquinolone		Glycylcyclines		colistin	fosfomycin
	cefoperazone + sulbactam	ceftolozane + tazobactam	cefotaxime	ceftazidime	ciprofloxacin	levofloxacin	tigecycline	minocycline		
CREC1	512 (R)	>1024 (R)	>1024 (R)	>1024 (R)	64 (R)	32 (R)	1 (R)	<2 (S)	0.5 (S)	16 (S)
CREC2	512 (R)	>1024 (R)	>1024 (R)	>1024 (R)	128 (R)	32 (R)	1 (R)	<2 (S)	2 (S)	16 (S)
CREC3	32 (S)	4 (S)	256 (R)	1024 (R)	4 (R)	<0.5 (S)	2 (R)	<2 (S)	1 (S)	64 (S)
CREC4	>1024 (R)	>1024 (R)	>1024 (R)	>1024 (R)	128 (R)	16 (R)	0.5 (S)	16 (R)	0.5 (S)	16 (S)
CREC5	>1024 (R)	>1024 (R)	>1024 (R)	>1024 (R)	128 (R)	16 (R)	1 (R)	<2 (S)	0.5 (S)	16 (S)
CREC6	512 (R)	>1024 (R)	>1024 (R)	>1024 (R)	0.5 (S)	<0.5 (S)	0.5 (S)	<2 (S)	0.5 (S)	16 (S)
CREC7	>1024 (R)	>1024 (R)	>1024 (R)	>1024 (R)	2 (I)	2 (S)	2 (R)	8 (I)	0.5 (S)	16 (S)
CREC8	>1024 (R)	>1024 (R)	>1024 (R)	>1024 (R)	4 (R)	1 (S)	2 (R)	4 (S)	2 (S)	16 (S)
CREC9	>1024 (R)	>1024 (R)	>1024 (R)	>1024 (R)	2 (I)	1 (S)	2 (R)	8 (I)	0.5 (S)	16 (S)
CREC10	>1024 (R)	>1024 (R)	>1024 (R)	>1024 (R)	64 (R)	32 (R)	1 (R)	4 (S)	1 (S)	16 (S)
CREC11	512 (R)	>1024 (R)	>1024 (R)	>1024 (R)	128 (R)	16 (R)	4 (R)	<2 (S)	0.25 (S)	16 (S)
CREC12	>1024 (R)	>1024 (R)	>1024 (R)	>1024 (R)	64 (R)	8 (R)	2 (R)	<2 (S)	0.25 (S)	16 (S)
CREC13	>1024 (R)	>1024 (R)	256 (R)	>1024 (R)	128 (R)	8 (R)	2 (R)	<2 (S)	0.5 (S)	16 (S)
CREC14	>1024 (R)	>1024 (R)	>1024 (R)	>1024 (R)	16 (R)	8 (R)	0.06 (S)	8 (I)	1 (S)	16 (S)
CREC15	256 (R)	>1024 (R)	>1024 (R)	>1024 (R)	128 (R)	8 (R)	0.25 (S)	<2 (S)	1 (S)	16 (S)

CREC16	512 (R)	>1024 (R)	>1024 (R)	>1024 (R)	256 (R)	32 (R)	2 (R)	16 (R)	1 (S)	32 (S)
CREC17	512 (R)	>1024 (R)	>1024 (R)	>1024 (R)	256 (R)	32 (R)	2 (R)	16 (R)	0.5 (S)	16 (S)
CREC18	512 (R)	>1024 (R)	>1024 (R)	>1024 (R)	512 (R)	64 (R)	2 (R)	<2 (S)	0.5 (S)	1024 (R)
CREC19	512 (R)	>1024 (R)	>1024 (R)	>1024 (R)	256 (R)	16 (R)	4 (R)	<2 (S)	1 (S)	1024 (R)

R, resistant; I, intermediate; S, susceptible.

Table S4. Summary of the synergistic effects of meropenem in combination with aminoglycosides against 19 carbapenem-resistant *Escherichia coli*.

Combination	Outcomes	
	Synergism (%)	Indifference (%)
meropenem + amikacin	13 (68.4)	6 (31.6)
meropenem + gentamicin	16 (84.2)	3 (15.8)
meropenem + kanamycin	15 (78.9)	4 (21.1)
meropenem + streptomycin	16 (84.2)	3 (15.8)
meropenem + tobramycin	15 (78.9)	4 (21.1)
